# Numerical Investigation of Effect of Nozzle Upper Divergent Angle on Asymmetric Rectangular Section Ejector

**DOI:** 10.3390/e27030312

**Published:** 2025-03-17

**Authors:** Manfei Lu, Jingming Dong, Chi Feng, Shuaiyu Song, Miao Zhang, Runfa Wang

**Affiliations:** Institute of Marine Engineering and Thermal Science, Marine Engineering College, Dalian Maritime University, Dalian 116026, China; lumanfei1120221216@dlmu.edu.cn (M.L.); fc11@dlmu.edu.cn (C.F.); dmussy@dlmu.edu.cn (S.S.); dmuzm@dlmu.edu.cn (M.Z.); wrf0128@dlmu.edu.cn (R.W.)

**Keywords:** asymmetric ejector, rectangular section, nozzle divergent angle, mixing layer, shock wave

## Abstract

Ejectors, as widely utilized devices in the field of industrial energy conservation, exhibit a performance that is significantly affected by their structural parameters. However, the study of the influence of nozzle geometry parameters on asymmetric ejector performance is still limited. In this paper, the effect of the nozzle upper divergent angle on the operating characteristics of an asymmetric rectangular section ejector was comprehensively investigated. The results indicated that the entrainment ratio gradually decreased with an increase in the nozzle upper divergent angle, and the maximum decrease could be 20%. At the same time, the relationship between the upper and lower divergent angles was closely linked to the trend of change in the secondary fluid mass flow rate. The analysis of flow characteristics found that the deflection of the central jet was caused by the pressure difference between the walls of the upper and lower divergent sections of the nozzle. Additionally, quantitative analysis of the development of the mixing layer showed that the mass flow rate of the secondary fluid inlet was related to the development of the mixing boundary. Shock wave analysis demonstrated that the deterioration in ejector performance was due to the reduction in the shock wave strength caused by Mach reflection and the increase in the Mach stem height.

## 1. Introduction

With the expansion of global large-scale social production, the consumption rate of non-renewable energy is increasing. This serious situation has prompted the innovation and rapid development of various energy-saving technologies. The main sections of an ejector are the nozzle, suction chamber, mixing chamber, constant-area mixing section, and diffuser. It is recognized as an efficient energy-saving device characterized by its simple structure and no additional power consumption. Therefore, the ejector is favored in the fields of seawater desalination [1], refrigeration [2], waste heat recovery [3], and adiabatic compressed air energy storage [4,5,6]. As computational fluid dynamics (CFD) continues to progress, many scholars [7,8,9,10,11] have adopted the CFD technique to carry out in-depth numerical studies on ejector performance.

The core of a supersonic jet predominantly occurs in the downstream region of the nozzle divergent section. Consequently, the structural parameters of this section significantly affect the ejector performance. Fu et al. [12] conducted a detailed investigation into the influence of the nozzle divergent length on the ejector performance. Their findings indicated that the entrainment ratio (*ω*) initially increases and then decreases with an increase in the nozzle divergent section length. Wang et al. [13] not only numerically investigated the nozzle divergent section length, but also optimized the nozzle divergent angle. They revealed that there are optimal parameters for both to obtain the best ejector performance. Similarly, Yan et al. [14] emphasized the importance of the nozzle divergent angle in improving ejector performance. Their research revealed that, under ideal operating conditions, the optimal angle can improve *ω* by 16.7%. Furthermore, Li et al. [15] used single-factor and multi-factor analysis methods to identify five critical structural parameters influencing ejector performance and sorted them according to their sensitivity. Among these parameters, the nozzle divergent angle was recognized as one of the most significant factors.

The internal mixing process between primary and secondary fluids is the main factor influencing an ejector. Numerous scholars have carried out investigations into this issue and achieved a series of remarkable research results. According to the standard one-dimensional theoretical model, the mixing process starts until the secondary flow is accelerated to the sonic level, and the two fluids flow independently in the first stage [16,17]. The mixing process in ejectors is the subject of ongoing research. Ariafar et al. [18] investigated the velocity vector distribution in the mixing chamber using numerical modeling. They discovered that the mixing layer first formed at the nozzle exit, where the interaction between primary and secondary fluids started. Subsequently, the mixing layer gradually thickened along the flow direction, and its thickness growth trend was approximately linear. Based on this discovery, they explored the relationship between the *ω* and the growth rate of the mixing layer. It was found that, with an increase in *ω*, the growth rate of the mixing layer accelerated accordingly. This is important for the optimization of ejector design [19]. Recently, Tang et al. [20] achieved the discrimination and tracing of primary and secondary fluids by introducing the species transport model into numerical simulations. Based on their results, they categorized the process of mixing layer growth into the following two stages: a fluctuating growth stage and an exponential growth stage. They also studied the influence law of operating conditions on the growth of the mixing layer and found that the influence of these conditions was more obvious during the exponential growth stage. Additionally, Tang et al. [21] provided a comprehensive comparative analysis of the evolution of the mixing layer from the perspectives of mass, momentum, and energy. This multi-dimensional perspective provides a new way to deeply understand the mechanism of the mixing process.

Complex processes, including shock wave formation and boundary layer separation, occur alongside supersonic fluid flow inside an ejector. These phenomena have a significant impact on ejector performance and have become the focus of research in this field. An analytical model was proposed by Zhu and Jiang [22] to predict the length of the first shock wave after the nozzle exit. They established a quantized relationship between the first shock length and *ω*, as follows: as the length of the first shock increased, the *ω* decreased. Similarly, Chen et al. [23] also focused on researching the shape parameter of the first shock wave inside the ejector. They found that the dimensionless height of the first shock wave increased with the primary fluid pressure and decreased with the secondary fluid pressure. This result can be attributed to the direct influence of shock wave height on the flow circulation of the secondary fluid. Arun et al. [24] studied a rectangular section ejector using numerical modeling in three dimensions. They noticed two different types of shock wave reflection laws occurring at the boundary layer within the mixing chambers. This discovery deepens researchers’ understanding of shock boundary layer reflection.

Although studies on ejectors have been widely explored, the current research trend is still mainly focused on symmetric structures. However, the existing studies show that under the same conditions, an asymmetric ejector is often superior to a symmetric ejector in some key indexes [25,26]. However, there are still relatively few studies on asymmetric ejectors, especially on key structural parameters such as the nozzle divergent angle. Current studies mostly focus on the effects of the pressure ratio, compression ratio, and temperature ratio of the primary and secondary flow on performance [27]. As for structural parameter studies of asymmetric rectangular section ejectors, particularly the impact of the nozzle upper divergent angle on their operating characteristics, these have not yet received sufficient attention. Therefore, this paper establishes a three-dimensional computational model of an asymmetric rectangular section ejector and employs the species transport equation to differentiate and calculate the mass fraction distribution of the fluid. In addition, the numerical Schlieren technique is used to present the specific form of the shock wave. This paper investigates the influence of the nozzle divergent angle on the performance, flow characteristics, mixing characteristics, and shock wave characteristics of the asymmetric rectangular section ejector. This paper reveals the mechanisms by which the nozzle upper divergent angle influences the ejector performance, thereby providing a theoretical basis for ejector design. By gaining a better understanding of how nozzle geometry parameters affect ejector performance, engineers can more effectively select and adjust nozzle designs to meet specific industrial requirements. Therefore, this study not only enhances the theoretical foundation of ejectors, but also offers valuable guidance for technological advancements and applications in related industries.

## 2. Methods

### 2.1. Geometry Model

**Figure 1:**
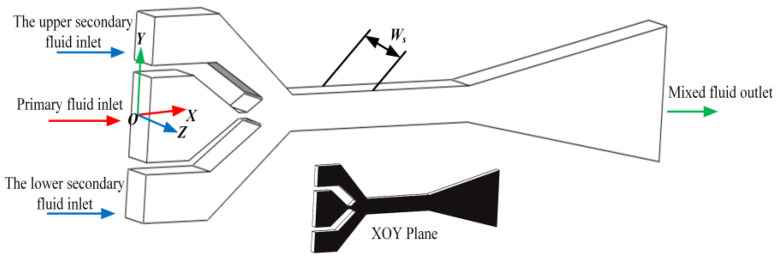
Three-dimensional structure diagram of the asymmetric rectangular section ejector.

Figure 1 displays the asymmetric rectangular section ejector studied in this paper. The ejector has two secondary fluid inlets, the upper secondary fluid inlet and the lower secondary fluid inlet. The mass flow of the secondary fluid is the sum mass flow rate of the lower secondary fluid inlet (*m_s_*_2_) and the upper secondary fluid inlet (*m_s_*_1_). The mass flow rate of the primary fluid inlet is (*m_g_*). Therefore, the entrainment ratio (*ω*) of the asymmetric rectangular section ejector in this paper is defined as follows.

(1) $$\omega =\frac{m_{s1}+m_{s2}}{m_{g}}$$

**Figure 2:**
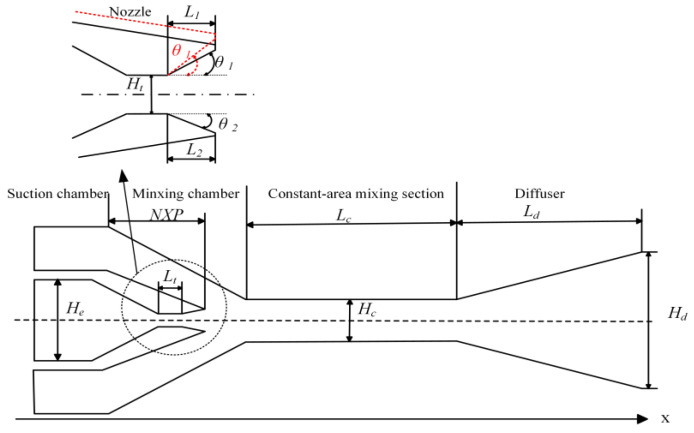
Structure diagram of the asymmetric rectangular section ejector on the XOY plane.

The axis of the ejector is taken as the reference line. As illustrated in Figure 2, the nozzle divergent angle consists of an upper divergent angle (*θ*_1_) and a lower divergent angle (*θ*_2_). The essence of the asymmetric rectangular section ejector described in this paper is to adjust the upper divergent angle only on the basis of the symmetrical structure, so as to give the ejector asymmetric characteristics. *θ*_1_ ranges from 9° to 30°, while *θ*_2_ remains constant at 18°. The structural characteristics of the asymmetric rectangular section ejector are referenced from Ref. [28]. The detailed dimensions are shown in Table 1.

### 2.2. Numerical Method and Physical Model

The following presumptions are introduced to simplify the numerical computation process:

(1) The fluid in the ejector is a compressible ideal gas.
(2) The walls of the ejector are non-slip and adiabatic.
(3) Throughout the entire operation, the temperature variations brought on by the gas supersonic movement are disregarded.
(4) The constant pressure principle governs the mixing process.
(5) At all inlets, the fluid velocity is disregarded.

The governing equation in the solving process was as follows.

Continuity equation:

(2) $$\frac{\partial \rho }{\partial t}+\frac{\partial }{\partial x_{i}}\left(\rho u_{i}\right)=0$$

Momentum equation:

(3) $$\frac{\partial }{\partial t}\left(\rho u_{i}\right)+\frac{\partial }{\partial x_{i}}\left(\rho u_{i}u_{j}\right)=-\frac{\partial P}{\partial x_{i}}+\frac{\partial \tau_{ij}}{\partial x_{j}}$$

Energy equation:

(4) $$\frac{\partial }{\partial t}\left(\rho E\right)+\frac{\partial }{\partial x_{i}}[u_{i}\left(\rho E+P\right)]=\vec{\nabla }\left(\alpha_{eff}\frac{\partial T}{\partial x_{i}}\right)+\vec{\nabla }\left[{u_{j}(\tau_{ij})}_{eff}\right]$$

(5) $$\tau_{ij}=\mu_{eff}\left(\frac{\partial u_{i}}{\partial x_{j}}+\frac{\partial u_{j}}{\partial x_{i}}\right)-\frac{2}{3}\mu_{eff}\frac{\partial u_{k}}{\partial x_{k}}\delta_{ij}$$

(6) $$\rho =\frac{P}{RT}$$

Many scholars have acknowledged the results of k-ω SST turbulence model simulations for supersonic ejectors [29,30]. Especially in numerical simulation calculations of rectangular section ejectors with air as the medium, k-ω SST model results are more consistent with experimental results compared to other turbulence models [24]. Therefore, the k-ω SST turbulence model is selected for calculation.

k-ω SST turbulence model equation:

(7) $$\bar{\rho u_{i}^{\prime }u_{j}^{\prime }}=\mu_{t}\left[\frac{\partial u_{i}}{\partial x_{j}}+\frac{\partial u_{j}}{\partial x_{i}}\right]-\frac{2}{3}\left[\rho k+\mu_{t}\frac{\partial u_{k}}{\partial x_{k}}\right]\delta_{ij}$$

(8) $$\frac{\partial }{\partial t}\left(\rho k\right)=\frac{\partial }{\partial x_{j}}\left[\Gamma_{k}\frac{\partial k}{\partial x_{j}}\right]+G_{K}-Y_{K}$$

(9) $$\frac{\partial }{\partial t}\left(\rho \omega \right)=\frac{\partial }{\partial x_{j}}\left[\Gamma_{\omega }\frac{\partial \omega }{\partial x_{j}}\right]+G_{\omega }-Y_{\omega }+D_{\omega }$$

The diffusion energy source model is enabled in the species transport model to mark primary and secondary flows for an intuitive study of mixing processes. The mass diffusion coefficient is set as 0 because the two types of air have identical characteristics. The species transport equation is as follows.

(10) $$\vec{\nabla }\left[\rho Y_{i}u\right]=\nabla \left[\rho D_{i,eff}\nabla Y_{i}\right]+S_{i}$$

where $Y_{N}=1-\sum_{i=1}^{N-1}Y_{i},D_{i,eff}=D_{i,m}+\frac{\mu }{\rho S_{ct}},S_{ct}=0.7$, $S_{i}=\dot{\omega }M_{w,i}$.

In this paper, Fluent is used as the solver of the CFD model, and the nonlinear control equations are discretized using a pressure-based solver. The coupled algorithm is chosen by the flow field iterative solution method to speed up the solution and improve its accuracy. The second-order upwind scheme is selected to complete the spatial discretization. The diffuser outlet is set as a “pressure outlet”, while the “pressure inlets” condition is applied to the inlets of primary and secondary fluids. The inlet pressure of the primary fluid is set as 500 kPa, the inlet pressure of the secondary fluid is set as 80 kPa, and the outlet pressure is set as 70 kPa to ensure that the ejector works during the critical working conditions. The standard temperature is 293 K. As the working medium of the ejector, air satisfies the gas state equation. Therefore, the fluid can be calculated as a continuous medium. The calculation is considered convergent when the residual value of the energy equation is less than 10^−6^ and the residual value of other variables is less than 10^−4^ [24].

### 2.3. Validation of Grid Independence

**Figure 3:**
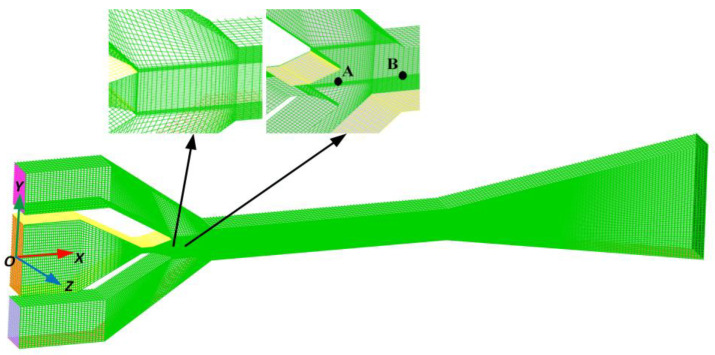
Three-dimensional grid diagram of the asymmetric rectangular section ejector.

The three-dimensional grid of the asymmetric rectangular section ejector is divided using ICEM CFD 2021 R1, as shown in Figure 3. The grid in the downstream region of the nozzle exit is locally refined to accurately capture the mixing process. To validate the grid independence, point A (the nozzle exit position at the axis) and point B (the center of the mixing chamber entrance at the axis) are chosen as the monitor data. Table 2 shows the calculation results and deviations of the two monitor points. It is evident from the table that the deviation of these points decreases with the grid number increasing from 280,636 to 609,964. Considering the calculation accuracy and calculation time, the number of grids used in this paper is not less than 430,396.

## 3. Results and Discussion

**Figure 4:**
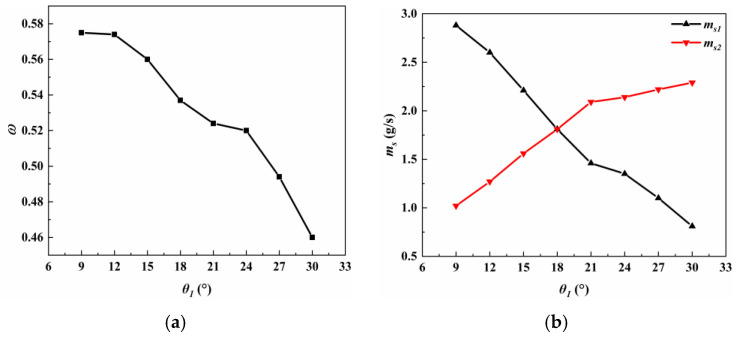
Variation in the asymmetric rectangular section ejector performance with different *θ*_1_: (**a**) entrainment ratio and (**b**) mass flow rate.

Figure 4 shows that the performance of the asymmetric rectangular section ejector varies with a varying *θ*_1_. As *θ*_1_ increases, ω decreases from 0.575 to 0.46, representing a reduction of 20%, as shown in Figure 4a. Notably, there is minimal variation in ω when *θ*_1_ increases from 9° to 12°. However, beyond this range, ω experiences a rapid decline. The most significant decrease in ω is observed when *θ*_1_ transitions from 24° to 30°. Figure 4b shows the change in the mass flow rate at different secondary fluid inlets of the asymmetric rectangular section ejector with *θ*_1_. From the figure, it can be seen that the variation trends of the mass flow rates of the two secondary fluid inlets of the asymmetric rectangular section ejector are different. Specifically, as *θ*_1_ increases, *m_s_*_1_ continues to decrease, while *m_s_*_2_ experiences an increase. With *θ*_1_ increasing from 9° to 18°, *m_s_*_1_ is more than *m_s_*_2_, while, with *θ*_1_ increasing from 18° to 30°, *m_s_*_2_ is more than *m_s_*_1_. In order to further study the variation trend of the entrainment performance of the asymmetric rectangular section ejector, the flow characteristics, mixing characteristics, and shock wave characteristics are analyzed in this paper.

### 3.1. Flow Characteristic Analysis

**Figure 5:**
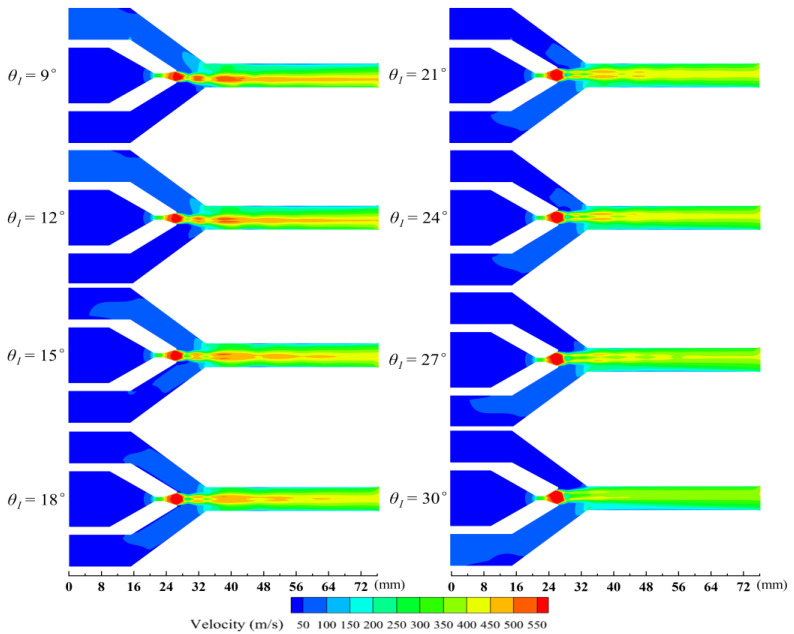
Velocity contours on the XOY plane with different *θ*_1_.

Figure 5 shows the velocity contours on the XOY plane of the asymmetric rectangular section ejector with different *θ*_1_. With an increase in *θ*_1_, the length of the central jet is gradually shortened and fluid separation gradually occurs. The velocity of the central jet of the primary fluid can reach a maximum of about 570 m/s. Additionally, the over-expansion of the primary fluid at the nozzle exit becomes more obvious with the increase in *θ*_1_, which restricts the ability of the primary fluid to entrain the secondary fluid. More importantly, the center jet of the primary fluid is deflected so that the flow area of the upper and lower secondary fluid in the constant-area section is changed. When *θ*_1_ is less than *θ*_2_, the central jet of the primary fluid is deflected downward, thereby making the flow area of the upper secondary fluid larger than that of the lower secondary fluid. Conversely, when *θ*_1_ is greater than *θ*_2_, the central jet is deflected upward, resulting in a reduced flow area for the upper secondary fluid relative to the lower secondary fluid. When these observations are considered alongside the results presented in Figure 4, it becomes evident that the mass flow rate of the secondary fluid is positively correlated with the flow area for the secondary fluid.

**Figure 6:**
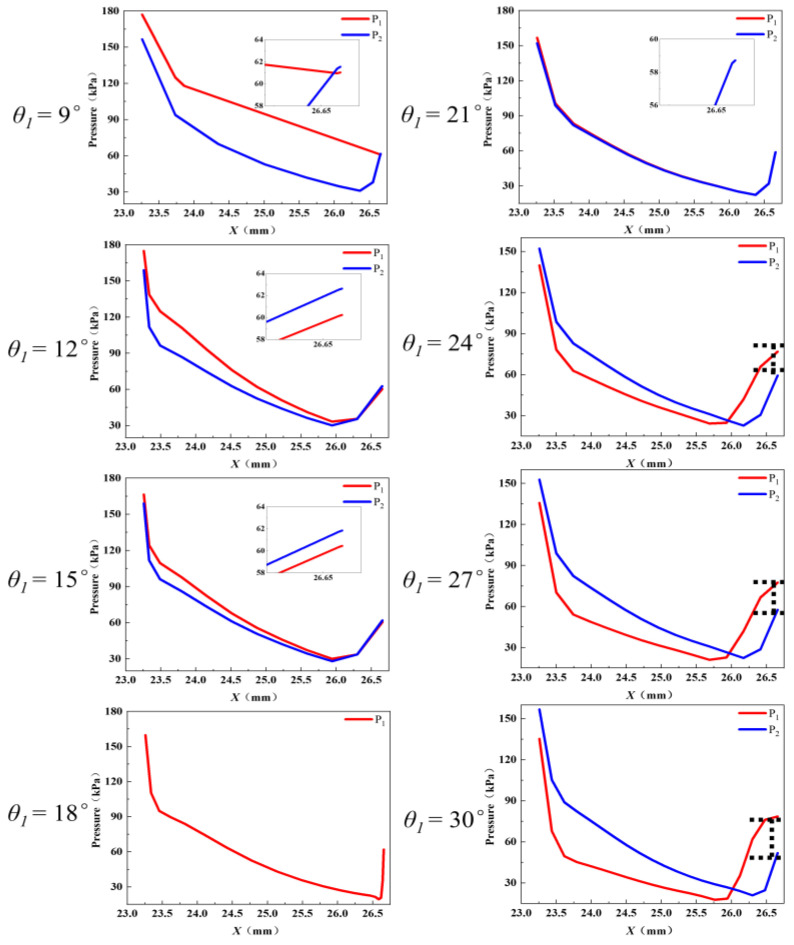
Wall pressure distribution in the nozzle divergent section with different *θ*_1_.

From Figure 5, it also can be observed that the deflection of the central jet originates from the nozzle divergent section. As the primary fluid enters this segment, the pressure energy is quickly transformed into kinetic energy. The primary fluid expands rapidly due to the sudden change in geometry, causing the pressure inside the nozzle divergent section to drop noticeably. Consequently, this study analyzes the wall pressure distribution in the nozzle divergent section. Figure 6 illustrates the wall pressure distribution along the nozzle divergent section with different *θ*_1_. When *θ*_1_ is smaller than *θ*_2_, the wall pressure on the nozzle upper divergent section (*P*_1_) is higher than that of the lower divergent section (*P*_2_), and then *P*_1_ is lower than *P*_2_ near the nozzle exit. Yet, where *θ*_1_ is greater than *θ*_2_, *P*_1_ is lower than *P*_2_, and *P*_1_ becomes higher than *P*_2_ near the nozzle exit. Thus, it can be assumed that the deflection of the central jet is caused by a pressure difference between the walls of the upper and lower divergent section close to the nozzle throat. The greater difference between *θ*_1_ and *θ*_2_, the larger pressure difference between the walls of the upper and lower divergent section near the nozzle throat, which results in a greater deflection of the central jet. Additionally, the wall pressure in the nozzle divergent section increases as it approaches the outlet, indicating that the primary and secondary fluids have begun to mix at this location.

### 3.2. Mixing Characteristic Analysis

**Figure 7:**
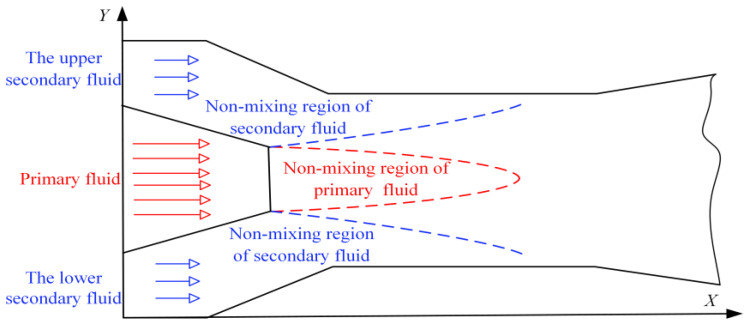
Schematic diagram of the formation of mixing layer within the ejector.

**Figure 8:**
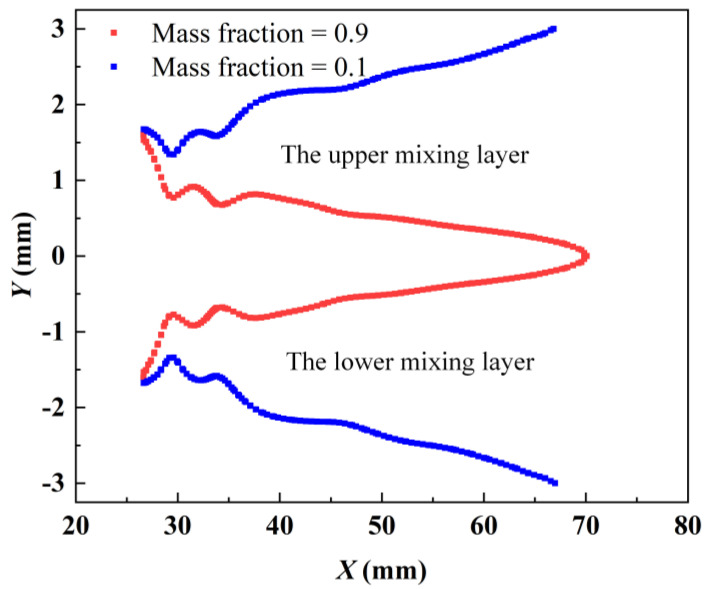
Schematic diagram of the upper and lower mixing layers of the rectangular section ejector.

The mixing characteristics of an ejector are a critical consideration in its design and application, especially in improving its efficiency and performance. Figure 7 presents a schematic diagram of the formation of the mixing layer within the ejector. Following its flow from the nozzle exit, the primary fluid interacts with the secondary fluid in the mixing chamber. Due to the large difference in the thermodynamic properties of the primary fluid and secondary fluid, when they come into contact, energy is exchanged, and eventually the two fluids mix completely and achieve equilibrium. This mixing process requires a transitional region in space, known as the mixing layer. The region outside of the mixing layer is referred to as the non-mixing region. The methodology used by Tang et al. [20] is adopted so that the mixing process can be comprehensively analyzed. Contours corresponding to primary fluid mass fractions of 0.9 and 0.1 are defined as the mixing boundaries between the primary fluid and secondary fluid. Based on the structural characteristics of the asymmetric rectangular section ejector, the mixing layers are categorized into the upper mixing layer and lower mixing layer to facilitate a more intuitive investigation of the mixing characteristics, as illustrated in Figure 8. The mass fraction of the secondary fluid boundary is 0.1, while the mass fraction of the primary fluid boundary is 0.9. The primary fluid boundary develops along the central axis of the ejector in a closed state, whereas the secondary fluid boundary gradually develops along the ejector wall.

**Figure 9:**
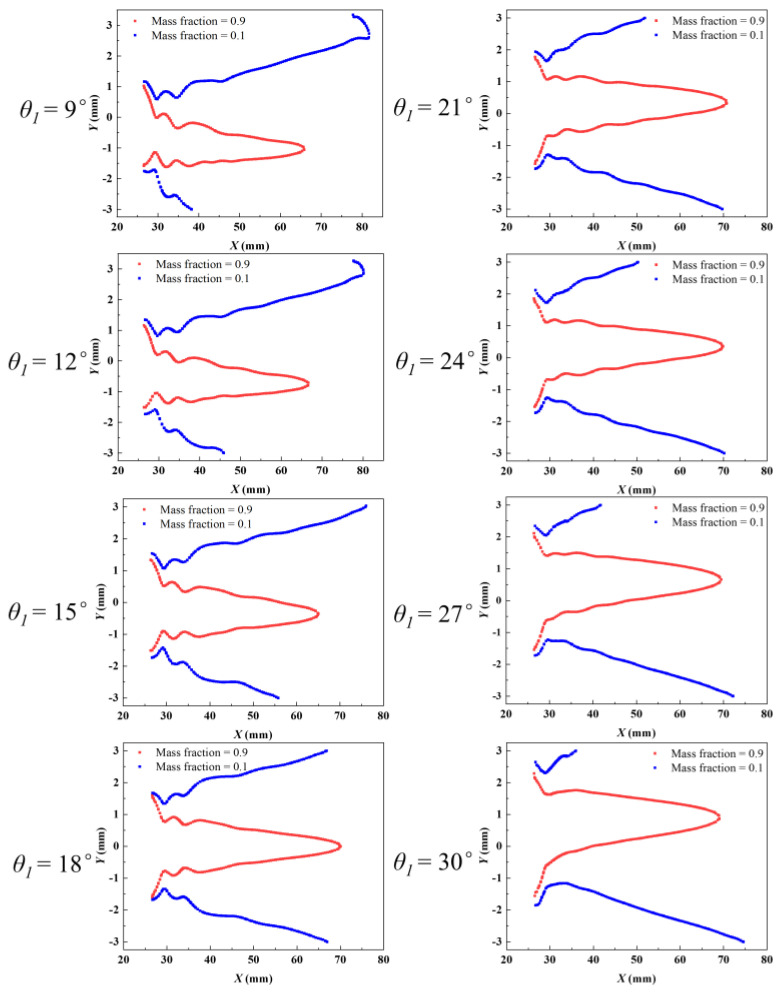
Development diagram of mixing boundaries with different *θ*_1_.

The development of the mixing boundaries with different *θ*_1_ is illustrated in Figure 9. The end positions of both the upper and lower mixing layer boundaries change with an increase in *θ*_1_. Specifically, as *θ*_1_ increases, the end positions of the primary fluid boundaries of the upper and lower mixing layers gradually shift back. This suggests that the primary fluid and secondary fluid are mixing less and less, that is, the primary fluid carries less and less secondary fluid. This is consistent with the results in Figure 4a. It is important to note that the regions closed by the primary fluid boundaries are not symmetrical. Specifically, when *θ*_1_ is smaller than *θ*_2_, the regions defined by the primary fluid boundaries are observed to be deflected downward. The primary fluid occupies the flow channels in the lower mixing layer, thus providing more regions for the flow channels in the upper mixing layer. Conversely, when *θ*_1_ is greater than *θ*_1_, the regions bounded by the primary fluid are deflected upward. In addition, as *θ*_1_ increases, the end position of the secondary fluid boundaries in the upper mixing layer is gradually shifted forward and the end position of the secondary fluid boundaries in the lower mixing layer is gradually shifted backward. For instance, the boundary of the secondary fluid in the upper mixing layer ends at *X* = 80.2 mm, while the boundary of the secondary fluid in the lower mixing layer ends at *X* = 38.8 mm when *θ*_1_ is set to 9°. Conversely, when *θ*_1_ is increased to 30°, the boundary of the secondary fluid in the upper mixing layer ends at an earlier position of *X* = 36.8 mm and the boundary of the secondary fluid in the lower mixing layer defers to a later position of *X* = 75.0 mm.

In order to further analyze the relationship between the mass flow rate and the secondary fluid boundaries, the following two evaluation indexes are introduced: the relative length of the secondary fluid boundary (*λ*) and the relative mass flow rate of the secondary fluid (*φ*). Since the asymmetric structure of the ejector is studied in this paper, it is necessary to analyze it in two cases, as follows.

(11) $$\lambda_{1}=\frac{l_{1}}{l_{f}}$$

(12) $$\lambda_{2}=\frac{l_{2}}{l_{f}}$$

where *λ*_1_ and *λ*_2_ represent the relative development lengths of the secondary fluid boundaries in the upper and lower mixing layers, respectively. *l*_1_ and *l*_2_ represent the lengths of the secondary fluid boundary from the nozzle exit to the ejector wall in the upper and lower mixing layers, respectively. *l_f_* is the length of the secondary fluid boundary from the nozzle exit to the ejector wall when *θ*_1_ is 18°.

(13) $$\varphi_{1}=\frac{m_{s1}}{m_{f}}$$

(14) $$\varphi_{2}=\frac{m_{s2}}{m_{f}}$$

where *φ*_1_ and *φ*_2_ represent the relative mass flow rates of the upper and lower secondary fluid inlets, respectively. *m_s_*_1_ and *m_s_*_2_ represent the mass flow rates of the upper and lower secondary fluid inlets. mf is the mass flow rate of the secondary fluid inlet when *θ*_1_ is 18°.

**Figure 10:**
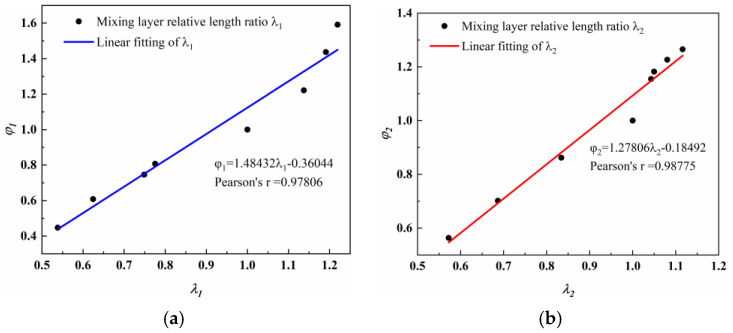
The function relationship between *φ* and *λ*: (**a**) (*φ*_1_, *λ*_1_) and (**b**) (*φ*_2_, *λ*_2_).

Figure 10 shows the relationship between the variables *φ* and *λ*. The data presented indicate that *φ* increases as *λ* rises. Furthermore, a strong linear correlation is observed between *φ*_1_ and *λ*_1_, as well as between *φ*_2_ and *λ*_2_. The Pearson’s correlation coefficients for these relationships are 0.97806 and 0.98775, respectively. The linear fitting function is provided as follows.

(15) $$\varphi_{1}=1.48432\lambda_{1}-0.36044$$

(16) $$\varphi_{2}=1.27806\lambda_{2}-0.18492$$

This significant linear correlation suggests that the full development of the mixing boundary has a critical influence on the mass flow rate of the secondary fluid inlet. The more adequate flow area for the secondary fluid facilitates a more complete development of the mixing boundary, thus allowing more secondary fluid to be entrained. This phenomenon can be interpreted as a higher value of *λ* indicating an increased contact area between the secondary and primary fluid, which subsequently enhances the probability of the secondary fluid being entrained by the primary fluid. Consequently, the value of *φ* increases in proportion to the value of *λ*.

### 3.3. Shock Wave Characteristic Analysis

The properties of the shock wave have a large impact on the ejector performance. This research examines the shock wave structure of the ejector using the numerical schlieren technique. The method effectively captures variations in the flow field density gradient, based on the principle that the density of the flow exactly correlates with the gradient of the refractive index. The orientation of the shock wave structure has a significant impact on the schlieren image quality. To enhance this image quality, Quirt [31] proposed using the absolute value of the density gradient. The mixing chamber and the downstream of the nozzle exit are identified as the two most critical locations for identifying shock waves. Therefore, these regions are locally refined during the meshing process, leading to a reduction in the grid cell volume in these areas. Based on this consideration, the present research refines Quirt’s formula for calculating shock wave intensity (*I*) by incorporating the grid cell volume (*β*) as a correction factor to improve the accuracy of the Schlieren image calculations. Here, *I* is defined as follows.

(17) $$I=\beta \left(\sqrt{{\left(\frac{\partial \rho }{\partial x}\right)}^{2}+{\left(\frac{\partial \rho }{\partial y}\right)}^{2}}\right)$$

**Figure 11:**
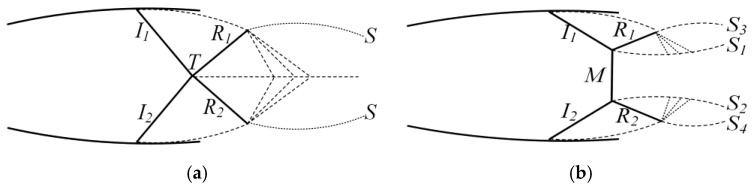
Schematic diagram of shock wave reflection: (**a**) regular reflection and (**b**) Mach reflection.

Ernst Mach’s investigations were the first to identify the occurrence of shock wave reflection [32]. The flow field properties of the shock wave are altered as a result of reflection. Therefore, the shock wave reflection significantly impacts the performance of the ejector. As illustrated in Figure 11, the shock wave reflection can be mainly categorized into the following two types: regular reflection and Mach reflection. Figure 11a shows the structure of a regular reflection shock. It consists of the incident shock waves (*I*_1_ and *I*_2_), the reflected shock waves (*R*_1_ and *R*_2_), the intersection point (*T*) of the incident shock wave and the reflected shock waves, and the boundary layer (*S*). Figure 11b shows the shock wave structure of Mach reflection. It consists of incident shock waves (*I*_1_ and *I*_2_), reflected shock waves (*R*_1_ and *R*_2_), and Mach stem (*M*), where *S*_1_ and *S*_2_ are the slip lines and *S*_3_ and *S*_4_ are the boundary layers.

**Figure 12:**
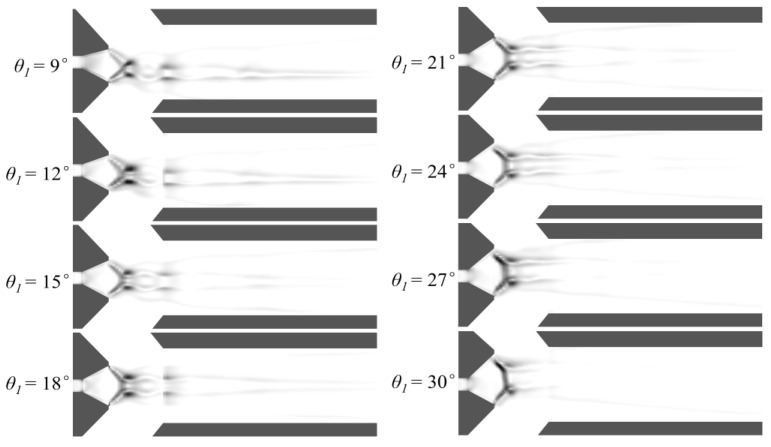
Numerical Schlieren images with different *θ*_1_.

Figure 12 presents numerical schlieren images with different *θ*_1_. Two compression waves as the first shock wave are observed after the nozzle exit for all structures. The difference is that these two compression waves are asymmetrically distributed due to the asymmetry of the nozzle divergent section, except for the structure with a *θ*_1_ of 18°. These two asymmetric shock waves intensify the deflection of the central jet. When *θ*_1_ is 9°, the two compression waves at the nozzle exit intersect to produce another compression wave. After, the compression waves pass through the fluid and are reflected as the expansion waves by the boundary layers. In this case, the first shock wave is characterized by regular reflection. As *θ*_1_ increases, this regular reflection turns into Mach reflection. As *θ*_1_ increases to 21°, the Mach stem becomes apparent. As *θ*_1_ is increased further, the height of the Mach stem steadily rises and the gap between the two slip lines widens. The region where the shock wave is located represents the flow region of the primary fluid. The increase in the height of the Mach stem leads to an increase in the region of the primary fluid in the Y-axis direction at the nozzle exit, thereby reducing the flow region of the nearby secondary fluid and restricting the secondary fluid being entrained. Moreover, the density gradient in the flow field decreases after Mach reflection. Especially when *θ*_1_ is 30°, there are no obvious compression and expansion waves in the numerical schlieren images. Combined with Figure 9, it can be seen that there is almost no fluctuation in the boundary of the primary fluid, which is not conducive to the mass transfer of the two fluids when *θ*_1_ is 30°. These two factors both lead to a deterioration in the ejector performance to some extent.

## 4. Conclusions

A three-dimensional mathematical model of an asymmetric rectangular section ejector was established in this paper. The effect of a nozzle upper divergent angle from 9° to 30° on the asymmetric rectangular section ejector performance was investigated. The reasons for the variation in performance of the asymmetric rectangular section ejector were explained in terms of flow characteristics, mixing characteristics, and shock wave characteristics. The following conclusions can be drawn:

(1) The nozzle upper divergent angle had a significant effect on the ω. With an increase in *θ*_1_, ω decreased gradually. The ω decreased from 0.575 to 0.46, a decrease of 20%. However, the variation trend of *m_s_*_1_ and *m_s_*_2_ was opposite. When *θ*_1_ was smaller than *θ*_2_, *m_s_*_1_ was greater than *m_s_*_2_. When *θ*_1_ was greater than *θ*_2_, *m_s_*_1_ was less than *m_s_*_2_.
(2) The deflection of the central jet of the primary fluid was caused by the pressure difference between the walls of the upper and lower expansion section of the nozzle.
(3) As *λ* increased, there was a corresponding increase in *φ*. There was a high degree of linear correlation between *λ* and *φ*.
(4) As *θ*_1_ increased, the reflection type of the first shock wave transitioned from regular reflection to Mach reflection. In the condition of Mach reflection, the increase in *θ*_1_ caused a rise in the height of the Mach stem, accompanied by a decrease in the intensity of the shock wave. These alterations collectively deteriorated the ejector performance to a certain extent.

## Figures and Tables

**Table 1:** Structure parameters of the asymmetric rectangular section ejector.

Parameter	Symbol	Value	Unit
Constant-area mixing section height	*H_c_*	6	mm
Diffuser outlet height	*H_d_*	22.3	mm
Nozzle entrance height	*H_e_*	14	mm
Nozzle throat height	*H_t_*	1	mm
Constant-area mixing section length	*L_c_*	42	mm
Diffuser length	*L_d_*	46.22	mm
Nozzle throat length	*L_t_*	2	mm
Nozzle upper divergent section length	*L*_1_	3.4	mm
Nozzle lower divergent section length	*L*_2_	3.4	mm
Ejector section width	*W_s_*	6	mm
Nozzle upper divergent angle	*θ*_1_	9, 12, 15, 18, 21, 24, 27, 30	°
Nozzle lower divergent angle	*θ*_2_	18	°
Nozzle exit position	*NXP*	11.41	°

**Table 2:** Grid independence test.

	Grid Numbers	Pressure (kPa)	Deviation (%)	Velocity (m/s)	Deviation (%)
Point A	280,636	21.497	0	595.47	0
	339,600	21.034	2.2012	594.12	0.2272
	389,840	20.747	1.3833	593.458	0.1116
	430,396	20.612	0.6549	593.276	0.0307
	522,148	20.712	0.4828	593.432	0.0263
	609,964	20.656	0.2711	593.521	0.0145
Point B	280,636	78.124	0	364.584	0
	339,600	79.911	2.2362	363.314	0.3500
	389,840	80.989	1.3310	362.922	0.1080
	430,396	80.247	0.9246	362.764	0.04355
	522,148	79.998	0.3113	362.740	0.0066
	609,964	79.841	0.1966	362.722	0.0050

## Data Availability

The research data supporting this publication are provided within this paper.
